# Supplementary material for: Learning Pelvic Anatomy and Pathology Through Drawing: An Interactive Session in the Obstetrics and Gynecology Clerkship
Source: MedEdPORTAL. 2023 Dec 5;19:11363. doi: 10.15766/mep_2374-8265.11363 (PMC10696139; doi:10.15766/mep_2374-8265.11363)
Supplement: Supplementary file 1 — Anatomy Presentation.pptxAnatomy Teacher Instructions.docxAnatomy Teaching Questions.docxAnatomy Teaching Questions with Answers.docxAnatomy Online Assessment.docxAnatomy Survey.docx [file mep_2374-8265.11363-s001.zip › _Educational Summary Report_11363.pdf]

MedEdPORTAL® | The AAMC Journal of Teaching and Learning Resources

Original Publication

Open Access

Learning Pelvic Anatomy and Pathology Through Drawing: An Interactive Session in the Obstetrics and Gynecology Clerkship

Elisabeth N. Adkins, MD, MBA*, Elizabeth Barrett, MD, Josette D'Amato, DO, Rose A. Maxwell, PhD, Marilyn Kindig, DO

*Corresponding author: enadkins95@gmail.com

Abstract

Introduction: Within undergraduate medical education, there is a gap between students' understanding of anatomy and application of that knowledge within surgical specialties. The integration of drawing, in conjunction with traditional learning, has been shown to increase retention and understanding of information. Currently, no educational curriculum integrates drawing to aid in medical students' understanding of surgical pelvic anatomy. We anticipated that the utilization of drawing anatomy in an OB/GYN clerkship would enhance students' ability to explain surgical pelvic anatomy and pelvic pathology. **Methods:** At the beginning of the OB/GYN clerkship, third-year medical students participated in an interactive, 1.5-hour session requiring them to draw pelvic anatomy, present their work, and explain topics related to pelvic surgery and pathology to the other clerkship students. At the end of their clinical rotation, the students were invited to complete a five-item survey to assess long-term retention and understanding of concepts presented in the session. Frequencies and percentages were calculated for all categorical/ordinal variables to describe survey participants and question responses. **Results:** Thirty-seven of 44 respondents (84%) reported that the anatomy interactive session prepared them for the surgical portion of the OB/GYN clinical rotation. Thirty-five respondents (80%) reported that drawing the pelvic structures helped their understanding of pelvic pathology; 33 respondents (75%) reported they had a thorough understanding of pelvic anatomy after taking the OB/GYN anatomy interactive educational session ($p < .001$). **Discussion:** Our session shows that integrating drawing and anatomy increases students' ability to discuss pelvic pathology and surgical anatomy.

Keywords
Anatomy, Drawing, Pelvic Pathology, Basic Science, OB/GYN, Pathology

Educational Objectives

By the end of this activity, participants will be able to:

1. Draw the anatomical structures of the pelvic region, including menstrual cycle and fetal blood circulation.
2. Discuss the correlation between pelvic anatomy and pathology.
3. Discuss the correlation between pelvic anatomy and OB/GYN surgical procedures.

Introduction

There is a known gap in undergraduate medical education between students' understanding of anatomy and their application of that knowledge within surgical specialties. This gap is specifically apparent in OB/GYN.[1] During the first 2 years of medical school, students are required to memorize anatomical structures without correlating them with their intraoperative clinical significance. Anatomy has historically been taught via problem-based learning, computer-assisted learning, lectures, and/or dissection.[2] Medical schools have transitioned from teacher-centered to learner-centered approaches, allowing students greater responsibility to acquire, learn, and retain information and to develop skills for lifelong learning. The generative drawing principle shows that people learn better from a scientific text when they are asked to draw illustrations representing the main idea of the text. Several studies have shown that asking students to draw pictures of didactic content improves deep cognition and metacognitive processing.[3] A study by Zhang and Fiorella compared learning from instructor-provided to learner-generated visuals in college students over the circulatory system.[4] The experiment showed that drawing was worth the added time, with an increase in cognitive load and a better performance on comprehensive exams.[4]

Citation:
Adkins EN, Barrett E, D'Amato J, Maxwell RA, Kindig M. Learning pelvic anatomy and pathology through drawing: an interactive session in the obstetrics and gynecology clerkship. *MedEdPORTAL.* 2023;19:11363. https://doi.org/10.15766/mep_2374-8265.11363

Copyright © 2023 Adkins et al. This is an open-access publication distributed under the terms of the Creative Commons Attribution license.

Further research is needed in this field, but high-fidelity medical simulations have already been shown to improve medical education.[5]

Despite these changes in approach, the gap remains between students' understanding of anatomy and application of that knowledge within surgical specialties. The integration of drawing in conjunction with traditional learning has been shown to increase retention and understanding of information.[6] Drawing requires students first to select verbal and visual information from presented materials and long-term memory, then to organize the information, and lastly to integrate it into a mental model that will be used to draw an external model.[5] When drawing is utilized to explain the function of more complex concepts, it reinforces the drawer's understanding of those concepts by enabling the drawer to visualize their thought process. As a result of this, students can simplify concepts that were unclear and deepen their current understanding. Furthermore, drawing assists students with spatial visualization, which is heavily influenced by anatomical understanding and is a strong predictor of surgical success.[7]

To date, when searching terms such as *anatomy, drawing, clerkship,* and *medical education,* no published educational curriculum is found that utilizes drawing to enhance medical students' understanding of surgical pelvic anatomy. Nor have we found any educational activities that integrate drawing anatomy within surgical clerkships. Furthermore, we have not seen any evaluations of drawing anatomy's correlation with medical students' understanding of surgical anatomy while in the operating room. The aim of our innovative teaching session was to implement the integration of drawing anatomy in an OB/GYN clerkship to enhance students' self-reported understanding of surgical pelvic anatomy and pelvic pathology. We anticipated that by using drawing to teach and reinforce surgical pelvic anatomy and physiology concepts, students would retain information longer and have a greater ability to explain surgical pelvic anatomy and pelvic pathology.

Methods

Session Design

The session was designed to increase third-year medical students' understanding of the pelvic anatomy and pathophysiology seen throughout their OB/GYN clerkship. We introduced the session with a presentation discussing the educational objectives and the seven structures that would be drawn (Appendix A). Structures varied from maternal-fetal circulation to the menstrual cycle. We selected which structures were drawn (Appendix B) based on clinical relevance and the likelihood that these topics would be seen within the operating room or that students would be asked questions about these topics while completing their clerkship. The session was taught by an attending OB/GYN faculty member and a fourth-year medical student who had shown proficiency in the topics. Fourth-year medical students were deemed proficient if they earned an 85% or higher OB/GYN clerkship grade, received above-average clerkship reviews by residents and faculty in the areas of fund of knowledge and professionalism, and/or received honors from the OB/GYN clerkship. This provided an opportunity for fourth-year medical students to improve their teaching and presentation skills, both of which were important for residency.

The interactive session was designed to take place either in person or virtually. It lasted approximately 1.5 hours and occurred during the first week of the OB/GYN clerkship. Third-year medical students were divided into groups of two to three from a larger group of 16-20 students. Each group was assigned one structure to draw, varying from the menstrual cycle to layers of the abdominal wall. If the session took place virtually, students utilized a videoconferencing platform where each group was assigned a breakout room. One student from each group drew their picture via Paint on Windows or Paintbrush on Mac or sent a hand-drawn copy to themselves via email. Group members who were not drawing were required to look up relevant information via web searches or anatomy books for their assigned structure and assist the group member who was drawing with labeling and verifying that the structure was drawn correctly. If the session took place in person, each group was given white poster-board paper and colored delible markers. All group members were required to work on the drawing and search for relevant information via web searches. Common websites utilized included Google, TeachMeAnatomy,[8] AMBOSS,[9] and UpToDate.[10] AMBOSS and UptoDate required a subscription to access content, while Google and TeachMeAnatomy did not.

Implementation

At the beginning of the session, a brief PowerPoint presentation was given that discussed the session outline, educational objectives, and requirements for each drawing (Appendix A). Students were then divided into groups of two to three and were given 30-35 minutes to draw their assigned structures. We supervised students' drawings and answered any questions students had related to the drawing or their understanding of their assigned topic's physiology or pathophysiology. After the allotted time, the groups presented their drawings. Each

Copyright © 2023 Adkins et al. This is an open-access publication distributed under the terms of the Creative Commons Attribution license.

group was given 3-4 minutes to explain its drawing (Figures 1 and 2). If the session was virtual, the group member who drew the structure was required to share their screen via the videoconferencing platform. We utilized predetermined questions for each assigned structure to ask each group member about basic physiology, pathology, or surgical anatomy related to their drawings (Appendices C and D). For instance, groups assigned to draw the abdominal wall layers were asked to give the name of the anterior wall muscles going into the fascia to sew two layers

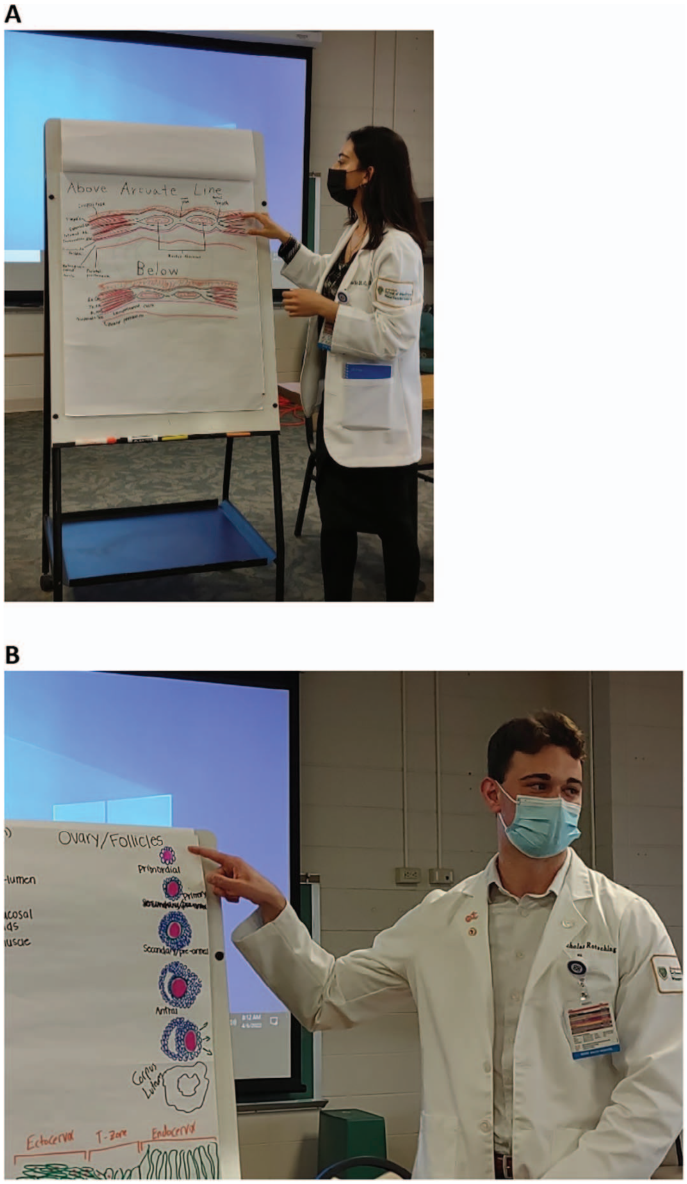

Figure 1. Medical students explaining their anatomy drawings to peers during the OB/GYN clerkship anatomy interactive session. A: Abdominal wall layers. B: The ovarian stages of ovulation.

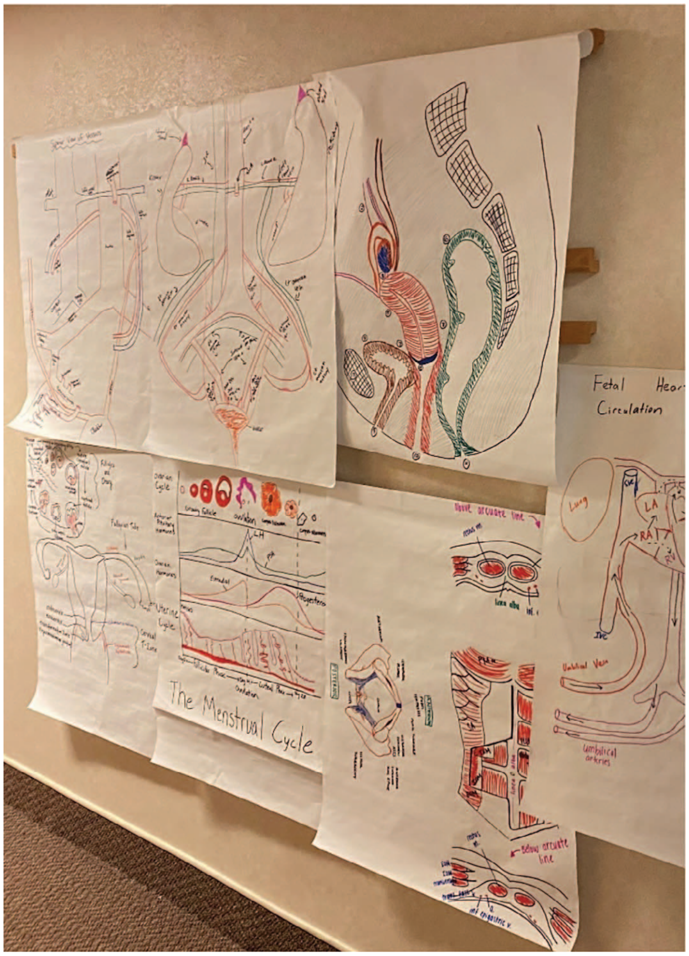

Figure 2. Images drawn by medical students for the OB/GYN clerkship anatomy didactic session.

closed during a cesarean section. If our fourth-year student instructor did not know the answer to a question posed by third-year medical students, the question was deferred to the attending faculty member.

Assessment

We created a required online assessment to test students' retention and understanding of the content learned in the interactive session (Appendix E). We curated the questions based on common themes that students found difficulty in explaining when asked by attendings or residents. These themes were related to topics discussed in the operating room, during morning rounds, or at other educational sessions. The questions were not piloted prior to being utilized for the anatomy interactive session.

Students were given 1 week after the anatomy interactive session to complete the online assessment. The assessment

Copyright © 2023 Adkins et al. This is an open-access publication distributed under the terms of the Creative Commons Attribution license.

opened at 12:00 AM Sunday and closed at 11:59 PM Saturday. It was administered remotely through the online software Elentra (Elentra Consortium), with a 1-hour duration for 16 questions. The assessment was open book; students were able to utilize internet search engines and textbooks to assist in answering questions.

During the last week of the clerkship, students completed an optional anonymous survey online to assess self-reported long-term retention and understanding of concepts from the anatomy interactive session (Appendix F). We developed survey items to assess students' self-reported understanding before and after the anatomy interactive session. Third-year medical students were chosen to complete the survey, as we believed this would provide a gauge from the learner perspective on how applicable the anatomy interactive session was for clinical encounters. The survey questions were not piloted prior to being utilized. The project was granted exemption by the university institutional review board for Wright State University Boonshoft School of Medicine.

Data were recorded electronically and analyzed with SPSS version 27.0 (IBM). Frequencies and percentages were calculated for all categorical/ordinal variables to describe participants and the main variables of interest (i.e., surgical pelvic anatomy session increasing understanding of OB/GYN surgical interventions and OB/GYN pathologies).

Results

There were 120 third-year medical students who participated in the anatomy interactive session. All students were required to complete the online assessment 1 week after finishing the session. The time at which the online assessment was available to students corresponded with the second week of the OB/GYN clinical rotation. The online assessment had a mean score of 88% and a median of 93% ($SD = 14\%$). Forty-four students (37% response rate) completed the voluntary survey during the final week of their OB/GYN clinical rotation, which was 6 weeks after the anatomy interactive session. Survey participants were third-year allopathic medical students who had never completed a clinical rotation in OB/GYN.

Most survey respondents reported that the anatomy interactive session prepared them for the surgical portion of the OB/GYN clinical rotation (84%) and that drawing the pelvic structures helped their understanding of pelvic pathology (80%; Table). When asked how they knew the drawing exercise had helped them, respondents chose "relationship/understanding" as the most frequent indication (50%). Eighteen percent of those who completed the optional survey reported that before taking the anatomy interactive session, they had a thorough understanding of pelvic anatomy; those same respondents did not find the anatomy interactive session helpful. Seventy-five percent of participants reported that they had a thorough understanding of pelvic anatomy after taking the OB/GYN anatomy interactive session ($p < .001$).

Table. Survey Questions Completed by Third-Year Medical Students ($N = 44$)

Question	No.	%
Thorough understanding of pelvic anatomy prior to the anatomy interactive session		
Yes	8	18
No	36	82
Thorough understanding of pelvic anatomy after the anatomy interactive session		
Yes	33	75
No	11	25
Anatomy interactive session prepared me for the surgical portions of the rotation		
Yes	37	84
No	7	16
Drawing the pelvic structures helped my understanding of pelvic pathology		
Yes	35	80
No	9	20
How do you know drawing pelvic structures has helped you with understanding pelvic pathology?		
Relationship/understanding	22	50
Visualize	8	18
Structure identification	2	5
Pimping	4	9
Not helpful	8	18

Students' comments regarding the anatomy interactive session included the following:

- "This was very influential for my learning. It made orientation more interactive. I found it incredibly useful, because it was a quick crash course on pelvic anatomy, something that I had learned in second year of medical school and forgotten about shortly after. If I have learned anything about preparing for a surgical rotation, it's 'know the anatomy', and this was a great refresher."
- "Being able to draw anatomical structures and use our own words to describe them and other concepts not only makes the concepts stick more clearly in our own minds, but it allows us to work on our communication in presentation skills and gives us a chance to learn from other peers."

Discussion

To address the knowledge gap in third-year medical students' ability to explain surgical pelvic anatomy and pathology, we developed an innovative interactive session. This session

Copyright © 2023 Adkins et al. This is an open-access publication distributed under the terms of the Creative Commons Attribution license.

utilized a learner-centered approach to engage students while integrating drawings to illustrate their thought process. Third-year medical students had a greater responsibility to acquire, learn, and verbalize the assigned information. Subsequently, more of them had a thorough self-reported understanding of information after completing the session.

It was evident that students understood the topics discussed within the interactive session from their ability to verbalize correlations between pelvic anatomy and pathology as well as to discuss the correlation of pelvic anatomy and surgical procedures throughout their OB/GYN clinical rotation.

The interactive session assisted in preparing students for various topics and procedures that would be encountered throughout their OB/GYN clinical rotation. It provided a safe, low-stakes environment for them to learn, verbalize, and discuss the correlation between pelvic anatomy, physiology, pathology, and surgical procedures. By secondary intention, students were also given an opportunity to improve their presentation skills while teaching and learning from their peers.

Our evaluation shows that learner-centered approaches can increase students' ability to explain surgical pelvic anatomy and pathology. Many survey respondents reported that the session increased their understanding of pelvic anatomy and pelvic pathology while preparing them for the surgical portions of their OB/GYN clinical rotation. Although 18% of medical students did not believe the session was helpful, these students already felt they understood pelvic anatomy. Students' survey responses were consistent with studies showing that integration of drawing with traditional learning techniques increases retention and understanding of information.[7,11,12] Although the online assessment was open book and was not an objective measurement of the effectiveness of the interactive session, students' comments and survey responses showed that the session increased their ability to discuss pelvic pathology and surgical anatomy. Our session is also consistent with the idea that learner-centered approaches where instruction is responsive, problem oriented, and democratic in nature lead to increased knowledge retention.[13,14]

Regarding instructing the session, no technical issues arose during the in-person session. Very few technical issues arose during the virtual session, and all were due to varying Wi-Fi speeds causing video or voice lag during students' presentations. When this occurred, another group member with a more stable internet connection presented the image. It is recommended that students with slow or unstable Wi-Fi be advised to travel to a location (e.g., school, hospital, coffee shop) with a more stable Wi-Fi connection. Prior to beginning the virtual session, we adjusted the web-meeting platform settings so that all participants were listed as presenters to ensure screen-sharing capabilities throughout the session.

The anatomy interactive session is feasible and provides opportunities for instructors to complete other clerical work. The session is designed for students to be more active in their learning, while instructors have a more subtle role compared to traditional teaching methods. This provides opportunities for instructors to attend to other tasks when not needed by students. For instance, during the 35 minutes allotted for drawing after all students' questions had been answered, we were able to complete other clerical tasks until it was time for students to discuss their drawings.

Our sample size of 44 students who completed the voluntary survey represented only 38% of third-year medical students who participated in the anatomy interactive session. This was likely due to the survey being voluntary and being disseminated during the final week of the OB/GYN clinical rotation when students had to complete exams, patient logs, and performance evaluations. The small sample size limited survey results, as it was not a full reflection of the opinions of all participants who completed the interactive session. The data were mostly likely skewed towards students who thoroughly enjoyed the interactive session, or the opposite. Moreover, the survey was given at the end of the 6-week clerkship, which increased recall bias.

In the future, to increase the survey response rate and minimize limitations, we recommend either assigning the survey an extra percentage point that counts toward clerkship participation or having students fill out the survey in person at the same time other clerkship clerical work is completed. It is further recommended that the weight of the extra percentage point not substantially influence the overall clerkship grade as the survey is voluntary. Alternatively, students could complete the survey at 3 weeks or mid-clerkship instead of during the final week. By mid-clerkship, students should have been exposed to the majority of the topics discussed within the interactive session, and recall bias should be less than during the final week of the clerkship. To objectively measure the effectiveness of the interactive session, we suggest making the online assessment closed book.

The information presented within the anatomy session focused primarily on surgical and pelvic pathology, topics only generally discussed during the first 2 years of medical education. All third-year medical students were required to complete the OB/GYN

Copyright © 2023 Adkins et al. This is an open-access publication distributed under the terms of the Creative Commons Attribution license.

anatomy interactive session to properly prepare for their clinical rotation. The evaluation of the interactive session was limited by one intervention being applied to all learners, with one postintervention survey. A randomized control trial could be utilized, with the control group having comparable interventions, such as assigned groups, topics, discussion, and answering questions, without the drawing portion of the anatomy interactive session.

It is evident from this interactive anatomy session that integrating drawing within the OB/GYN clerkship enhances students' ability to discuss pelvic physiology, pathology, and surgical procedures. This session is innovative, feasible, and learner centered. Although novel, we believe it can be incorporated across other surgical specialties, such as plastic surgery, trauma surgery, and general surgery, to increase learner engagement and knowledge retention of clinically relevant anatomy.

Appendices

A. Anatomy Presentation.pptx

B. Anatomy Teacher Instructions.docx

C. Anatomy Teaching Questions.docx

D. Anatomy Teaching Questions with Answers.docx

E. Anatomy Online Assessment.docx

F. Anatomy Survey.docx

All appendices are peer reviewed as integral parts of the Original Publication.

Elisabeth N. Adkins, MD, MBA: First-Year Resident, Department of Obstetrics and Gynecology, Vanderbilt University Medical Center; ORCID: https://orcid.org/0000-0002-6026-701X

Elizabeth Barrett, MD: Fourth-Year Resident, Department of Obstetrics and Gynecology, Wright State University Boonshoft School of Medicine

Josette D'Amato, DO: Assistant Professor, Department of Obstetrics and Gynecology, Wright State University Boonshoft School of Medicine

Rose A. Maxwell, PhD: Associate Professor and Research Director, Department of Obstetrics and Gynecology, Wright State University Boonshoft School of Medicine

Marilyn Kindig, DO: Assistant Professor and Clerkship Director, Department of Obstetrics and Gynecology, Wright State University Boonshoft School of Medicine

Disclosures

None to report.

Funding/Support

None to report.

Ethical Approval

The Wright State University Boonshoft School of Medicine Institutional Review Board deemed further review of this project not necessary.

References

1. Bergman EM, de Bruin ABH, Herrler A, Verheijen IWH, Scherpbier AJJA, van der Vleuten CPM. Students' perceptions of anatomy across the undergraduate problem-based learning medical curriculum: a phenomenographical study. *BMC Med Educ*. 2013;13:152. https://doi.org/10.1186/1472-6920-13-152
2. Singh V, Kharb P. A paradigm shift from teaching to learning gross anatomy: meta-analysis of implications for instructional methods. *J Anat Soc India*. 2013;62(1):84-89. https://doi.org/10.1016/s0003-2778(13)80019-6
3. Leutner D, Schmeck A. The generative drawing principle in multimedia learning. In: Mayer RE, ed. *The Cambridge Handbook of Multimedia Learning*. 2nd ed. Cambridge University Press; 2014:433-448.
4. Zhang Q, Fiorella L. Learning by drawing: when is it worth the time and effort? *Contemp Educ Psychol*. 2021;66:101990. https://doi.org/10.1016/j.cedpsych.2021.101990
5. Issenberg SB, Mcgaghie WC, Petrusa ER, Gordon DL, Scalese RJ. Features and uses of high-fidelity medical simulations that lead to effective learning: a BEME systematic review. *Med Teach*. 2005;27(1):10-28. https://doi.org/10.1080/01421590500046924
6. Heisler CA. Importance of adequate gross anatomy education: the impact of a structured pelvic anatomy course during gynecology fellowship. *Anat Sci Educ*. 2011;4(5):302-304. https://doi.org/10.1002/ase.235
7. Quillin K, Thomas S. Drawing-to-learn: a framework for using drawings to promote model-based reasoning in biology. *CBE Life Sci Educ*. 2015;14(1):es2. https://doi.org/10.1187/cbe.14-08-0128
8. TeachMeAnatomy. Accessed October 20, 2023. https://teachmeanatomy.info/
9. AMBOSS. Accessed October 20, 2023. https://www.amboss.com/us
10. UpToDate. Accessed October 20, 2023. https://www.wolterskluwer.com/en/solutions/uptodate
11. Na Y, Clary DW, Rose-Reneau ZB, et al. Spatial visualization of human anatomy through art using technical drawing exercises. *Anat Sci Educ*. 2022;15(3):587-598. https://doi.org/10.1002/ase.2080

Copyright © 2023 Adkins et al. This is an open-access publication distributed under the terms of the Creative Commons Attribution license.

12. Borrelli MR, Leung B, Morgan MCB, Saxena S, Hunter A. Should drawing be incorporated into the teaching of anatomy? *J Contemp Med Educ*. 2018;6(2):34-48.

13. Weimer M. *Learner-Centered Teaching: Five Key Changes to Practice*. 2nd ed. Jossey-Bass; 2013.

14. Dupin-Bryant PA. Teaching styles of interactive television instructors: a descriptive study. *Am J Distance Educ*. 2004;18(1): 39-50. https://doi.org/10.1207/s15389286ajde1801_4

Received: January 31, 2023
Accepted: September 8, 2023
Published: December 5, 2023

Copyright © 2023 Adkins et al. This is an open-access publication distributed under the terms of the Creative Commons Attribution license.
