# Supplementary material for: Learning Pelvic Anatomy and Pathology Through Drawing: An Interactive Session in the Obstetrics and Gynecology Clerkship
Source: MedEdPORTAL. 2023 Dec 5;19:11363. doi: 10.15766/mep_2374-8265.11363 (PMC10696139; doi:10.15766/mep_2374-8265.11363)
Supplement: Supplementary file 1 — Anatomy Presentation.pptxAnatomy Teacher Instructions.docxAnatomy Teaching Questions.docxAnatomy Teaching Questions with Answers.docxAnatomy Online Assessment.docxAnatomy Survey.docx [file mep_2374-8265.11363-s001.zip › A. Anatomy Presentation.pptx]

## Slide 1
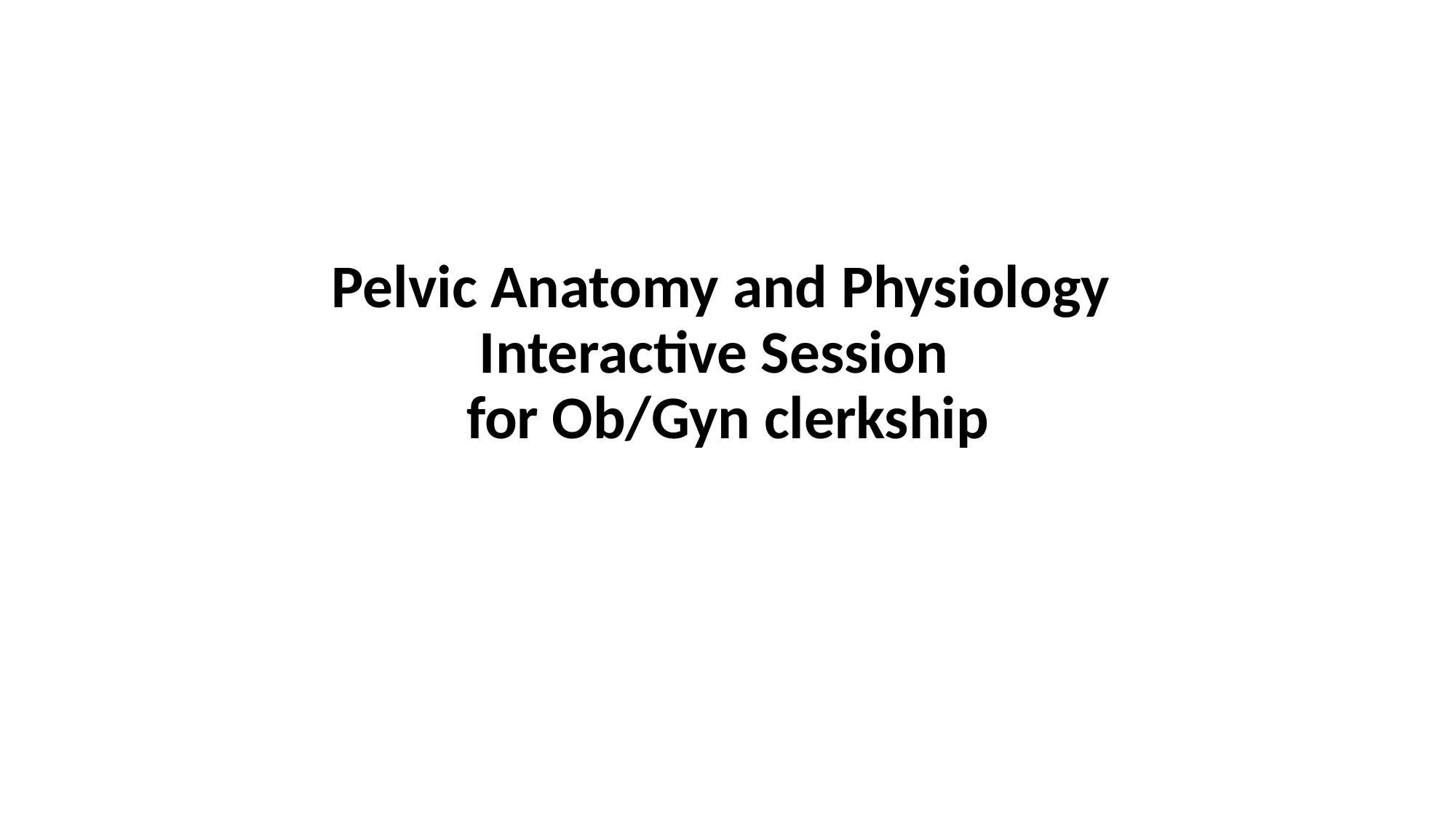

# Pelvic Anatomy and Physiology Interactive Session   for Ob/Gyn clerkship

## Slide 2
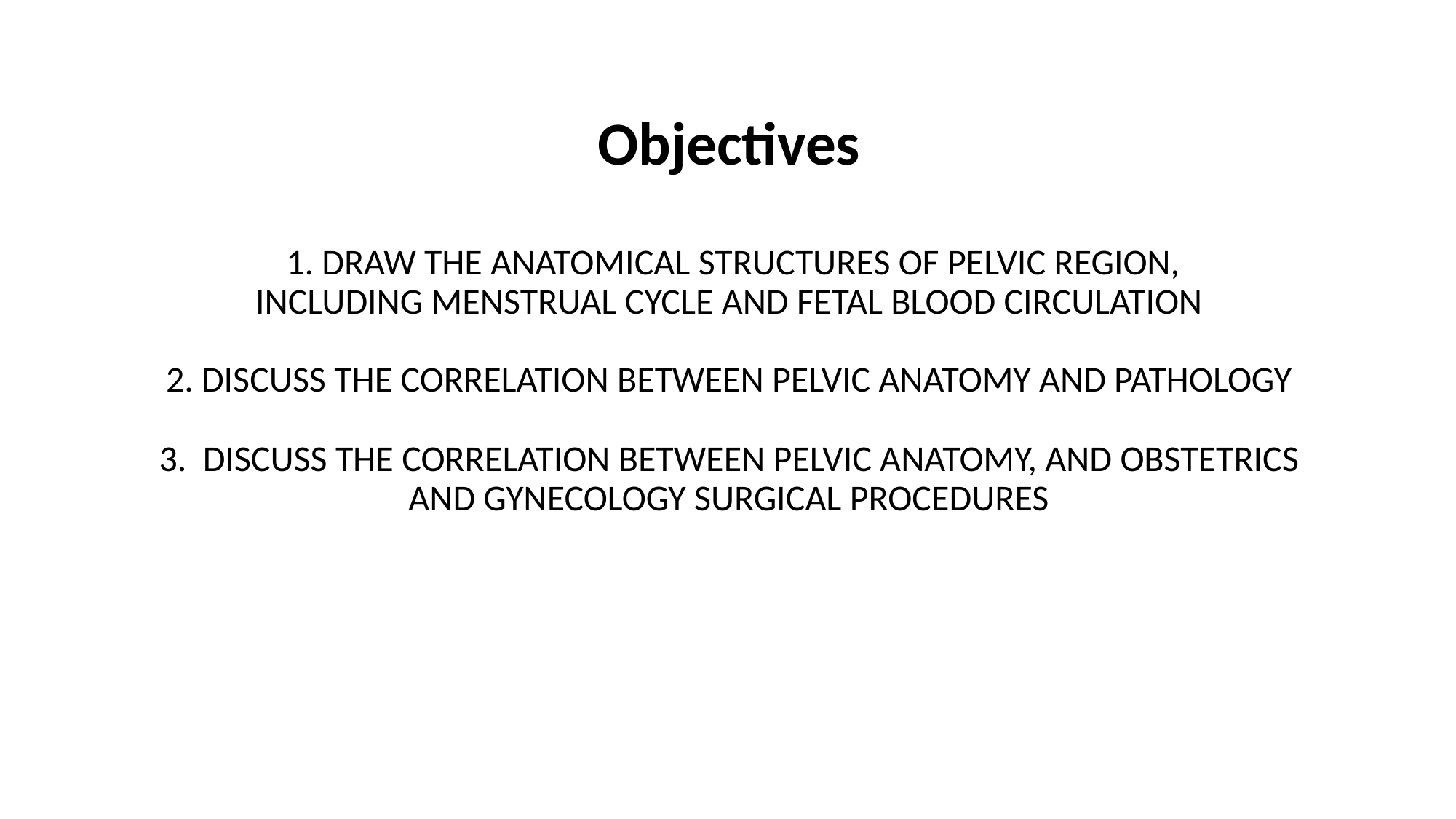

# Objectives  1. DRAW THE ANATOMICAL STRUCTURES OF PELVIC REGION, INCLUDING MENSTRUAL CYCLE AND FETAL BLOOD CIRCULATION 2. DISCUSS THE CORRELATION BETWEEN PELVIC ANATOMY AND PATHOLOGY 3.  DISCUSS THE CORRELATION BETWEEN PELVIC ANATOMY, AND OBSTETRICS AND GYNECOLOGY SURGICAL PROCEDURES

## Slide 3
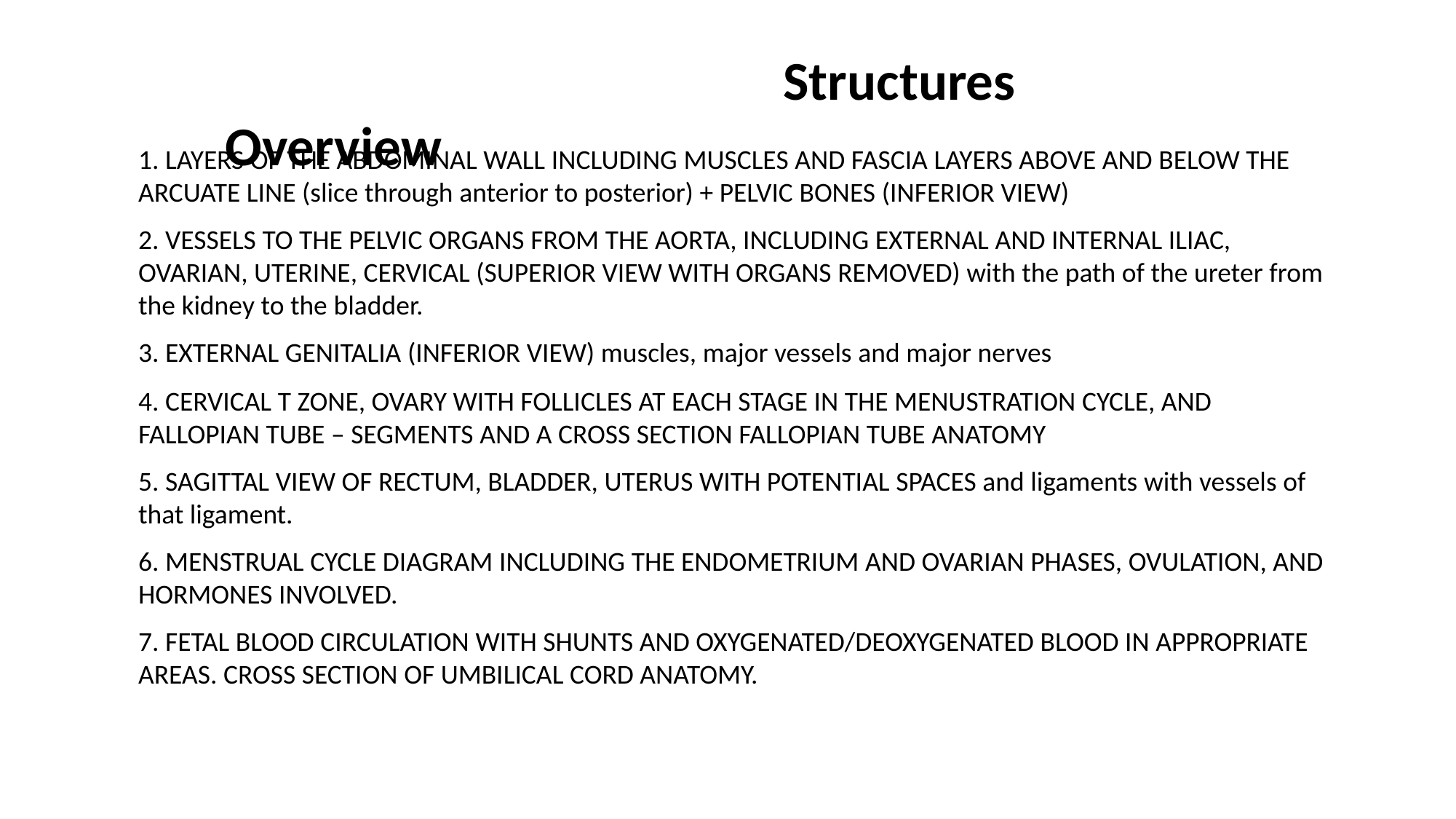

Structures Overview
#
1. LAYERS OF THE ABDOMINAL WALL INCLUDING MUSCLES AND FASCIA LAYERS ABOVE AND BELOW THE ARCUATE LINE (slice through anterior to posterior) + PELVIC BONES (INFERIOR VIEW)
2. VESSELS TO THE PELVIC ORGANS FROM THE AORTA, INCLUDING EXTERNAL AND INTERNAL ILIAC, OVARIAN, UTERINE, CERVICAL (SUPERIOR VIEW WITH ORGANS REMOVED) with the path of the ureter from the kidney to the bladder.
3. EXTERNAL GENITALIA (INFERIOR VIEW) muscles, major vessels and major nerves
4. CERVICAL T ZONE, OVARY WITH FOLLICLES AT EACH STAGE IN THE MENUSTRATION CYCLE, AND FALLOPIAN TUBE – SEGMENTS AND A CROSS SECTION FALLOPIAN TUBE ANATOMY
5. SAGITTAL VIEW OF RECTUM, BLADDER, UTERUS WITH POTENTIAL SPACES and ligaments with vessels of that ligament.
6. MENSTRUAL CYCLE DIAGRAM INCLUDING THE ENDOMETRIUM AND OVARIAN PHASES, OVULATION, AND HORMONES INVOLVED.
7. FETAL BLOOD CIRCULATION WITH SHUNTS AND OXYGENATED/DEOXYGENATED BLOOD IN APPROPRIATE AREAS. CROSS SECTION OF UMBILICAL CORD ANATOMY.

## Slide 4
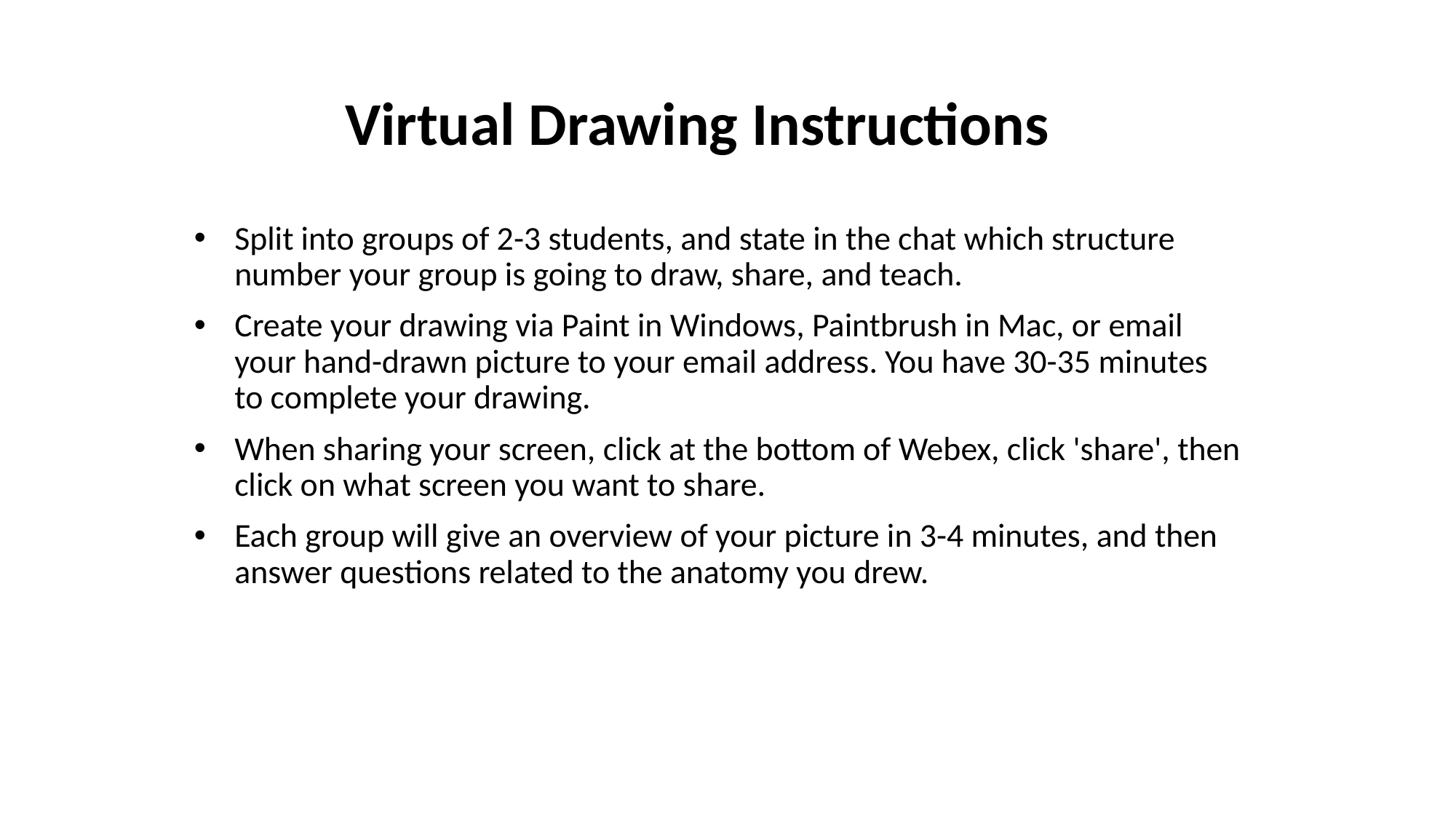

# Virtual Drawing Instructions
Split into groups of 2-3 students, and state in the chat which structure number your group is going to draw, share, and teach.
Create your drawing via Paint in Windows, Paintbrush in Mac, or email your hand-drawn picture to your email address. You have 30-35 minutes to complete your drawing.
When sharing your screen, click at the bottom of Webex, click 'share', then click on what screen you want to share.
Each group will give an overview of your picture in 3-4 minutes, and then answer questions related to the anatomy you drew.

## Slide 5
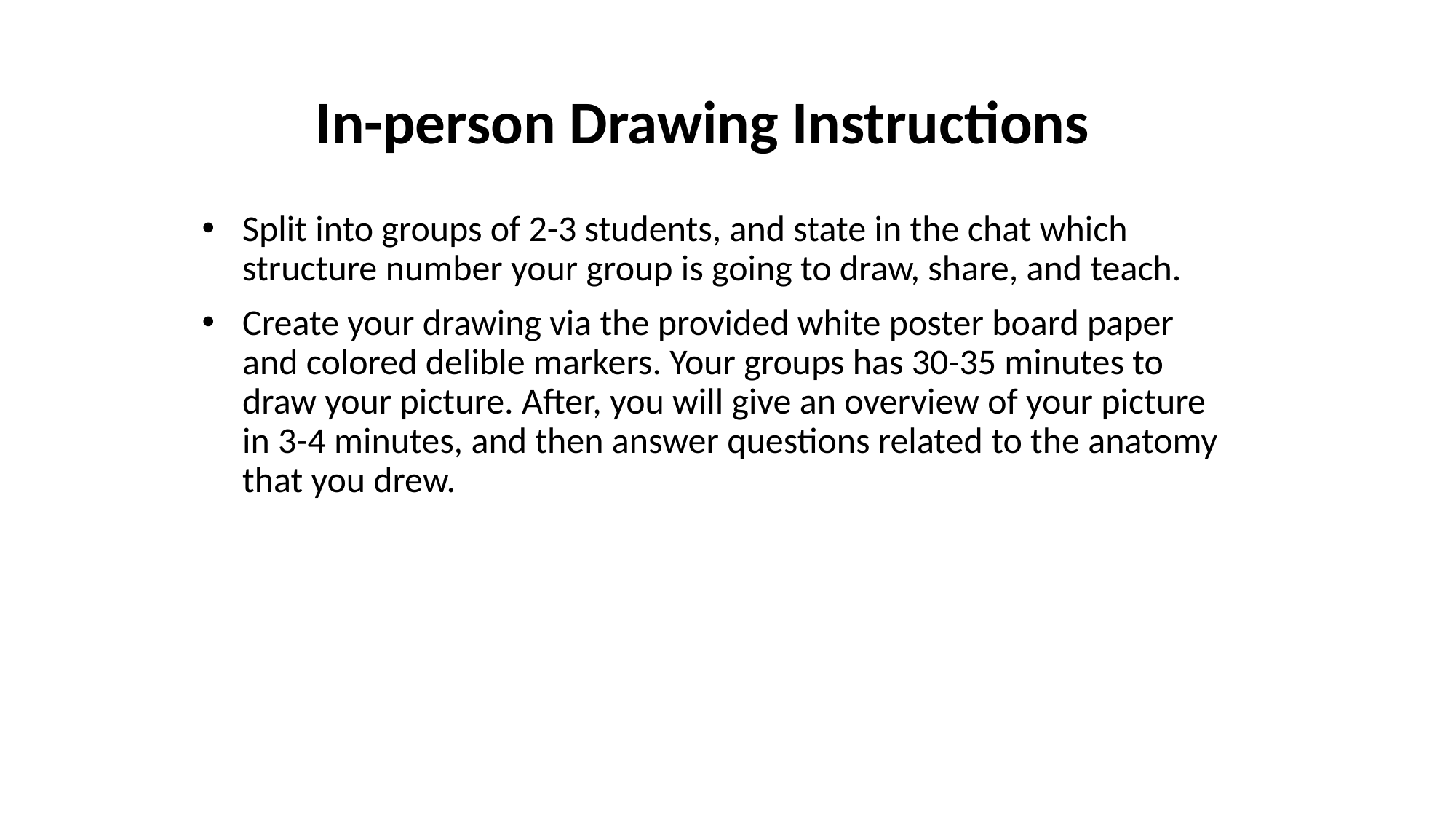

# In-person Drawing Instructions
Split into groups of 2-3 students, and state in the chat which structure number your group is going to draw, share, and teach.
Create your drawing via the provided white poster board paper and colored delible markers. Your groups has 30-35 minutes to draw your picture. After, you will give an overview of your picture in 3-4 minutes, and then answer questions related to the anatomy that you drew.

## Slide 6
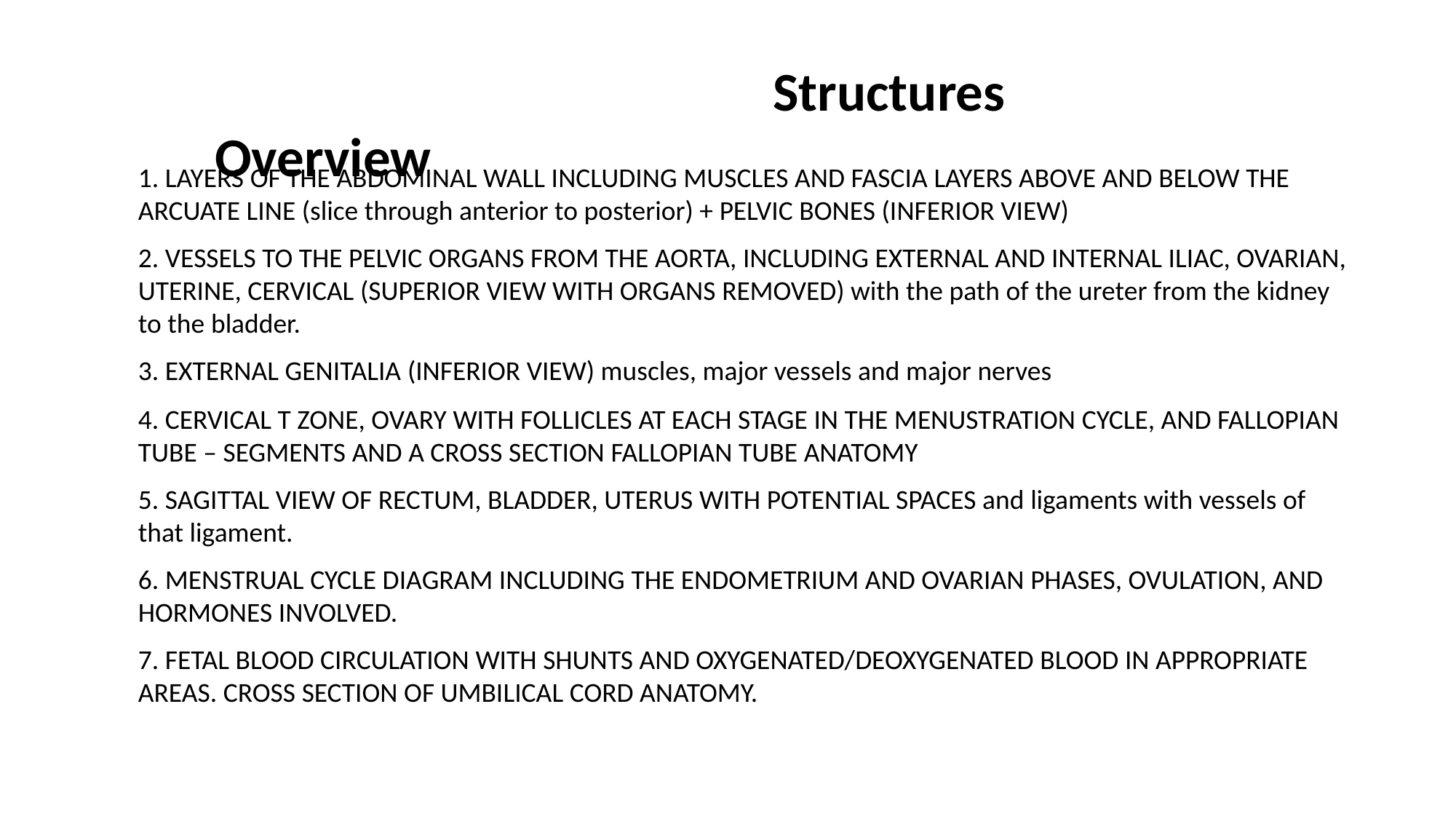

Structures Overview
#
1. LAYERS OF THE ABDOMINAL WALL INCLUDING MUSCLES AND FASCIA LAYERS ABOVE AND BELOW THE ARCUATE LINE (slice through anterior to posterior) + PELVIC BONES (INFERIOR VIEW)
2. VESSELS TO THE PELVIC ORGANS FROM THE AORTA, INCLUDING EXTERNAL AND INTERNAL ILIAC, OVARIAN, UTERINE, CERVICAL (SUPERIOR VIEW WITH ORGANS REMOVED) with the path of the ureter from the kidney to the bladder.
3. EXTERNAL GENITALIA (INFERIOR VIEW) muscles, major vessels and major nerves
4. CERVICAL T ZONE, OVARY WITH FOLLICLES AT EACH STAGE IN THE MENUSTRATION CYCLE, AND FALLOPIAN TUBE – SEGMENTS AND A CROSS SECTION FALLOPIAN TUBE ANATOMY
5. SAGITTAL VIEW OF RECTUM, BLADDER, UTERUS WITH POTENTIAL SPACES and ligaments with vessels of that ligament.
6. MENSTRUAL CYCLE DIAGRAM INCLUDING THE ENDOMETRIUM AND OVARIAN PHASES, OVULATION, AND HORMONES INVOLVED.
7. FETAL BLOOD CIRCULATION WITH SHUNTS AND OXYGENATED/DEOXYGENATED BLOOD IN APPROPRIATE AREAS. CROSS SECTION OF UMBILICAL CORD ANATOMY.
