# Supplementary material for: Learning Pelvic Anatomy and Pathology Through Drawing: An Interactive Session in the Obstetrics and Gynecology Clerkship
Source: MedEdPORTAL. 2023 Dec 5;19:11363. doi: 10.15766/mep_2374-8265.11363 (PMC10696139; doi:10.15766/mep_2374-8265.11363)
Supplement: Supplementary file 1 — Anatomy Presentation.pptxAnatomy Teacher Instructions.docxAnatomy Teaching Questions.docxAnatomy Teaching Questions with Answers.docxAnatomy Online Assessment.docxAnatomy Survey.docx [file mep_2374-8265.11363-s001.zip › B. Anatomy Teacher Instructions.docx]

Appendix B: Anatomy teacher instructions

Appendix B: Anatomy Teacher Instructions

*Utilize this appendix to determine materials needed for virtual or in-person session. Roles and responsibilities remain the same for instructors regardless of setting, but slightly differ for technology assistant based on whether the session is in-person or online. Estimated time needed to complete the session remains the same regardless of location at approximately 1.5 hr.*

Roles & Responsibilities:

1. Instructor:
   1. Giving presentation prior to students beginning drawing
   2. Assigning students into groups and giving them drawing assignments
   3. Assisting students during their drawing
   4. Facilitating discussion about questions and answers of each group assigned topic during group presentations
2. Technology assistant:
   1. Ensuring computer and screen projector are working
   2. Distributing supplies to each group
   3. If session is facilitated online – creating and dispersing video conference link to students and instructor
   4. If session is facilitated online – ensure that students have access to ‘share’ their screen throughout web conference link by making everyone ‘presenter’

Materials needed:

1. Instructor:
   1. In person:
      1. Computer with internet access
      2. Projector
   2. Online:
      1. Computer with internet access, webcam, and microphone
2. per group:
   1. In-person
      1. Colored markers
      2. Post-its super sticky easel pad, 25 x 30 inches
      3. Computer with internet
   2. Online
      1. Computer with internet and webcam
      2. Copier paper, 8.5 x 11 inches
      3. Pen or pencils
      4. ii & iii can be substituted with paintbrush (Apple) or Paint (Windows)- only one student

Estimated timing for anatomy interactive session:

Total – 1 hr. 30 minutes

1. PowerPoint Presentation – 5-7 minutes
2. Assigning groups and drawing pictures – 30 - 35 minutes
3. Group presentation: 3-4 minutes per group
4. Questions: 5-7 minutes per group

Drawing topics:

- - - 1. Layers of the abdominal wall including muscles and fascia layers above and below the arcuate line (slice through anterior to posterior) + pelvic bones (inferior view).
      2. Pelvic organs vessels from the aorta, including external and internal iliac, ovarian, uterine, cervical with the path of the ureter from the kidney to the bladder.
      3. External genitalia (inferior view) muscles, major vessels, and major nerves.
      4. Cervical T zone, ovary with follicles at each stage in the menstruation cycle and fallopian tube – segments and a cross section fallopian tube anatomy fallopian tube anatomy. ***

4a) cervical T zone, ovaries with follicles at each stage within menstruation cycle.

4b) fallopian tube – segments and a cross section fallopian tube anatomy, the uterus with endometrial layers, potential fibroid positions.

1. Sagittal view of rectum, bladder, uterus with potential spaces and ligaments with vessels within those ligaments.
2. Menstrual cycles diagram including endometrium and ovarian phases, ovulation, and hormones involved.
3. Fetal blood circulation with shunts and oxygenated/deoxygenated blood in appropriate areas, plus cross section of umbilical cord anatomy. ***

****Structures that are assigned to groups of students can be modified based on the size of the clerkship class, adding a group by splitting #4 or eliminating #7****
