# Supplementary material for: Learning Pelvic Anatomy and Pathology Through Drawing: An Interactive Session in the Obstetrics and Gynecology Clerkship
Source: MedEdPORTAL. 2023 Dec 5;19:11363. doi: 10.15766/mep_2374-8265.11363 (PMC10696139; doi:10.15766/mep_2374-8265.11363)
Supplement: Supplementary file 1 — Anatomy Presentation.pptxAnatomy Teacher Instructions.docxAnatomy Teaching Questions.docxAnatomy Teaching Questions with Answers.docxAnatomy Online Assessment.docxAnatomy Survey.docx [file mep_2374-8265.11363-s001.zip › C. Anatomy Teaching Questions.docx]

**Appendix C: Anatomy teaching questions**

Appendix C: Anatomy Teaching Questions

*** Utilize these questions to facilitate discussion about each group’s drawing. Groups should be given 3-4 minutes to explain their drawing. Allocate 5-7 minutes per group to answer the questions related to their drawing. If students do not know the answer, explain the answer to them and its relationship to their drawing. Questions can be modified based on what medical students will see throughout their clerkship rotation and/or areas of interest or knowledge gaps that commonly arise throughout the session. Total time for this portion of interactive session: approximately 40-50 minutes***

**#1: Layers of the abdominal wall including muscles and fascia layers above and below the arcuate line (slice through anterior to posterior) + pelvic bones (inferior view)**

- What are the names of the muscles of the anterior abdominal wall that go into the fascia to sew two layers when closing a cesarean section incision?
- What are the vessels that perforate through the anterior abdominal wall muscles and are at risk of bleeding during laparoscopy trocar placement?
- What is the name of the aponeurosis of the transversus abdominis, external oblique, and internal oblique muscles?
- What hormone helps relax the pelvic bones during labor?
- Where is the ischial spine and why is it important? Name the procedure and the other pelvic structure that the ischial spine serves as a landmark for.
- Which foramen contains the obturator canals? Which surgery are they important for?

**#2: Pelvic organs vessels from the aorta, including external and internal iliac, ovarian, uterine, cervical with the path of the ureter from the kidney to the bladder**

- Which vessel do the ovarian arteries arise from?
- Where do each of the ovarian veins drain?
- In relation to the broad ligament, is the ureter on the medial or lateral side?
- What is the order of the thigh/pelvic vessels from lateral to medial of the thigh?
  - Which vein is in the popliteal fossa? What should a patient in a hypercoagulable state be evaluated for if they present with pain within the calf or popliteal fossa?
- Explain the course of the ureters.

**#3: External genitalia (inferior view) muscles, major vessels, and major nerves**

- Where do you find the urethra in relation to clitoris?
- Where is the most common place to get a cyst at 4 or 8 o’clock on the perineum?
- How do you incise a Bartholin’s cyst?
- What are the female homologues from embryology of the male's penis and scrotum?
- From embryology, how do two vaginas or two uteri happen?
- If you have a uterine absence or duplication, what other system should you check?
- Where and why is the perineal body so important?
  - How would a patient present if they experienced an internal and external anal sphincter muscle injury?

**#4a: Cervical T zone, ovary with follicles at each stage in the menstruation cycle, and fallopian tube – segments and a cross section fallopian tube anatomy fallopian tube anatomy**

**4a: cervical T zone, ovary with follicles at each stage in the menstruation cycle**

- What is the transformation zone?
- Why is the transformation zone important in high-risk HPV such as 16, 18, 31, 33?
- What portion of the cervix does a cervical conization remove? What is cervical conization utilized for?
- Describe the different stages of the ovarian follicles during the menstrual cycle.
  - What is the most common type of ruptured ovarian cyst, and when does it most often occur? How may a patient present?

**4b: fallopian tube – segments and a cross section fallopian tube anatomy fallopian tube anatomy**

- Name each portion of the fallopian tube? What is the importance of cilia in fertilization?
- Which structures are removed in a tubal ligation?
- What is the nomenclature for an ectopic pregnancy within the horns of the uterus?
- What are the three layers within the endometrium?
- What are the types of fibroids (leiomyoma) within the uterus and their locations?

**#5: Sagittal view of rectum, bladder, uterus with potential spaces and ligaments with vessels within those ligaments**

- What is the relationship of the ligaments to the uterus, cervix, vagina, ovaries? (Broad ligament, round ligament of the uterus, cardinal ligament, ovarian ligament, infundibulopelvic ligament)
- What is the artery to the round ligament called?
- What is the ligament that carries the ovarian artery and vein?
- What is the parasympathetic and sympathetic suppl of the uterus and cervix? What is the innervation of the pelvis and ovaries?
- What is innervation of the pelvic diaphragm?
- What is the pelvic potential spaces nomenclature?
  - Describe the different areas where fibroids can occur, and which ones can impede getting pregnant.

**#6: Menstrual cycles diagram including endometrium and ovarian phases, ovulation, and hormones involved**

- Per Association of Professors of Gynecology & Obstetrics (APGO), what is the length of a normal cycle?
- What is the nomenclature for the first part of the ovarian phase and endometrium phase? What is the primary hormone released from the anterior pituitary and ovaries/corpus luteum during this time?
- What is the nomenclature for the second part of the ovarian phase and endometrium phase? What is the primary hormone released from the anterior pituitary and corpus luteum during this time?
- What hormone surge causes ovulation to occur?
- How long is the luteal phase?
- How long is the follicular phase?
- When does the corpus luteum stop producing progesterone when pregnancy occurs?

**#7: Fetal blood circulation with shunts and oxygenated/deoxygenated blood in appropriate areas, plus cross section of umbilical cord anatomy**

- Name the three active shunts in utero and then their adult remnants.
- What happens to the umbilical artery after birth?
- Which important physiological changes occur to the lungs at delivery and its impact on blood circulation?
