# Supplementary material for: Learning Pelvic Anatomy and Pathology Through Drawing: An Interactive Session in the Obstetrics and Gynecology Clerkship
Source: MedEdPORTAL. 2023 Dec 5;19:11363. doi: 10.15766/mep_2374-8265.11363 (PMC10696139; doi:10.15766/mep_2374-8265.11363)
Supplement: Supplementary file 1 — Anatomy Presentation.pptxAnatomy Teacher Instructions.docxAnatomy Teaching Questions.docxAnatomy Teaching Questions with Answers.docxAnatomy Online Assessment.docxAnatomy Survey.docx [file mep_2374-8265.11363-s001.zip › D. Anatomy Teaching Questions with Answers.docx]

**Appendix D: Anatomy Teaching Questions with Answers**

Appendix D: Anatomy Teaching Questions with Answers

*** Utilize these questions to facilitate discussion about each group’s drawing. Groups should be given 3-4 minutes to explain their drawing. Allocate 5-7 minutes per group to answer the questions related to their drawing. If students do not know the answer, explain the answer to them and its relationship to their drawing. Questions can be modified based on what medical students will see throughout their clerkship rotation and/or areas of interest or knowledge gaps that commonly arise throughout the session. Total time for this portion of interactive session: approximately 40-50 minutes***

**#1: Layers of the abdominal wall including muscles and fascia layers above and below the arcuate line (slice through anterior to posterior) + pelvic bones (inferior view)**

- What are the names of the muscles of the anterior abdominal wall that go into the fascia to sew two layers when closing a cesarean section incision?

*Internal and external oblique fascia, below the arcuate line.*

- What are the vessels that perforate through the anterior abdominal wall muscles and are at risk of bleeding during laparoscopy trocar placement?

*Inferior epigastric vessels.*

- What is the name of the aponeurosis of the transversus abdominis, external oblique, and internal oblique muscles?

*Rectus sheath.*

- What hormone helps relax the pelvic bones during labor?

*Relaxin.*

- Where is the ischial spine and why is it important? Name the procedure and the other pelvic structure that the ischial spine serves as a landmark for.

*Lateral to mid-sacrum; it is important for the pudendal nerve block and to palpate the sacrospinous ligament for location of ischial spines.*

- Which foramen contains the obturator canals? Which surgery are they important for?

*Obturator foramen, connecting the pelvis to the medial compartment of the thigh; trans-obturator sling procedure for refractory stress incontinence and pelvic prolapse.*

**#2: Pelvic organs vessels from the aorta, including external and internal iliac, ovarian, uterine, cervical with the path of the ureter from the kidney to the bladder**

- Which vessel do the ovarian arteries arise from?

*Abdominal aorta.*

- Where do each of the ovarian veins drain?

*Left ovarian vein drains into left renal vein, right ovarian vein drains into inferior vena cava.*

- In relation to the broad ligament, is the ureter on the medial or lateral side?

*Medial leaf of broad ligament.*

- What is the order of the thigh/pelvic vessels from lateral to medial of the thigh?

*Femoral nerve, femoral artery, femoral vein, lymph nodes.*

- - Which vein is in the popliteal fossa? What should a patient in a hypercoagulable state be evaluated for if they present with pain within the calf or popliteal fossa?

*Popliteal vein; deep venous thrombosis.*

- Explain the course of the ureters.

*Renal pelvis, over common iliac arteries, under uterine arteries.*

**#3: External genitalia (inferior view) muscles, major vessels, and major nerves**

- Where do you find the urethra in relation to clitoris?

*First opening under the clitoris.*

- Where is the most common place to get a cyst at 4 or 8 o’clock on the perineum?

*Bartholin cyst duct located within the vulva.*

- How do you incise a Bartholin’s cyst?

*Marsupialization or incision and drainage with the placement of a word catheter.*

- What are the female homologues from embryology of the male's penis and scrotum?

*Clitoris male homologue is penis; labia majora male homologue is scrotum.*

- From embryology, how do two vaginas or two uteri happen?

*Failure of fusion of Mullerian ducts.*

- If you have a uterine absence or duplication, what other system should you check?

*Kidneys and ureters for urinary Mullerian anomalies with renal imaging.*

- Where and why is the perineal body so important?

*Central tendon of the perineum and essential for the integrity of the pelvic floor; perineal ruptures, which are common in childbirth predisposes to pelvic organ prolapse.*

- - How would a patient present if they experienced an internal and external anal sphincter muscle injury?

*Difficulty to feel stool or gas for elimination; internal anal sphincter is involuntary smooth muscle;* it *will not be associated with pain; external anal sphincter is voluntary skeletal muscle and will be*  *associated with pain.*

**#4a: Cervical T zone, ovary with follicles at each stage in the menstruation cycle, and fallopian tube – segments and a cross section fallopian tube anatomy fallopian tube anatomy**

**4a: cervical T zone, ovary with follicles at each stage in the menstruation cycle**

- What is the transformation zone?

*It is the transition point between the endocervix and ectocervix, also known as the squamocolumnar junction.*

- Why is the transformation zone important in high-risk HPV such as 16, 18, 31, 33?

*It is most susceptible to HPV infection and carcinogenesis, possibly due to immortalization and dysplastic differentiation.*

- What portion of the cervix does a cervical conization remove? What is cervical conization utilized for?

*Transformation zone and a portion of the endocervical canal; utilized for definitive of squamous or glandular intraepithelial lesions and management of cervical intraepithelial neoplasia.*

- Describe the different stages of the ovarian follicles during the menstrual cycle.

*Ovarian follicles began as primary. Follicles begin to mature into secondary follicles due to follicular stimulating hormone, which leads to the development of the dominant follicle (Graafian follicle). Estrogen eventually reaches a peak, which causes a luteinizing hormone (LH) surge. The LH surge causes ovulation of an oocyte from the graafian follicle, corresponding with the luteal phase of the ovaries. The Graafian follicle now becomes the corpus luteum to produce progesterone. This release of the oocyte to development of the corpus luteum is the time that a hemorrhagic cyst may develop.*

- - What is the most common type of ruptured ovarian cyst, and when does it most often occur? How may a patient present?

*A hemorrhagic cyst is the most common type of ruptured ovarian cyst.* *From the release of the oocyte to development of the corpus luteum is the time that a hemorrhagic cyst may develop. A patient may present with sudden, sharp, lower quadrant pain with or without vaginal bleeding or spotting. Imaging will show free fluid in the peritoneal cavity around the ovaries, indicative of a ruptured cyst.*

**4b: fallopian tube – segments and a cross section fallopian tube anatomy fallopian tube anatomy**

- Name each portion of the fallopian tube? What is the importance of cilia in fertilization?

*From uterus to ovary: intramural, isthmus, ampulla, infundibulum, and fimbriae; the cilia capture the oocyte after release from ovulation and is necessary for fertilization.*

- Which structures are removed in a tubal ligation?

*Removal of entire fallopian tube due to postulation of fallopian tube leading irritating peritoneum leading to ovarian cancer.*

- What is the nomenclature for an ectopic pregnancy within the horns of the uterus?

*Cornual ectopic pregnancy.*

- What are the three layers within the endometrium?

*Stratum compactum, stratum spongiosum and stratum basalis, with the first two being the functional layer of the endometrium (stratum functionalis).*

- What are the types of fibroids (leiomyoma) within the uterus and their locations?

*Submucous, intramural, subserosal and pedunculated. Submucosal is within the myometrium just below the endometrial lining; intramural is within the uterine corpus wall; subserosal is on the exterior surface of the uterus beneath the serosa; pedunculated can be submucosal or subserosal.*

**#5: Sagittal view of rectum, bladder, uterus with potential spaces and ligaments with vessels within those ligaments**

- What is the relationship of the ligaments to the uterus, cervix, vagina, ovaries? (Broad ligament, round ligament of the uterus, cardinal ligament, ovarian ligament, infundibulopelvic ligament)

*The broad ligament attaches the lateral uterus, fallopian tubes, and the ovaries to the lateral pelvic wall, composed of mesovarium, mesometrium, and mesosalpinx.*

*The round ligament of uterus connects uterine horn to labia majora, running within the inguinal canal.*

*The cardinal ligament connects the cervix to the lateral pelvic wall.*

*The ovarian ligament attaches the medial pole of the ovary to the lateral uterus horn.*

*The infundibulopelvic ligament attaches the ovaries to the lateral pelvic wall and contains the ovarian artery and vein.*

- What is the artery to the round ligament called?

*The artery to the round ligament or Sampson’s artery.*

- What is the ligament that carries the ovarian artery and vein?

*Infundibulopelvic ligament or suspensory ligament.*

- What is the parasympathetic and sympathetic suppl of the uterus and cervix? What is the innervation of the pelvis and ovaries?

*Uterus: parasympathetic – pelvic splanchnic nerves; sympathetic - inferior hypogastric plexus. Cervix: parasympathetic - uterovaginal plexus; sympathetic: inferior hypogastric plexus.*

*Vagina: pudendal nerve.*

*Ovary: ovarian nerve plexus along ovarian artery and superior ovarian nerve in suspensory ligament.*

- What is innervation of the pelvic diaphragm?

*Pudendal nerves (S2-S4).*

- What is the pelvic potential spaces nomenclature?

*Anterior and posterior cul-de-sac, rectouterine pouch, vesicouterine pouch.*

- Describe the different areas where fibroids can occur, and which ones can impede getting pregnant.

*Fibroids may be intramuscular, subserosal, submucosal or pedunculated; submucosal may impede implantation of zygote.*

**#6: Menstrual cycles diagram including endometrium and ovarian phases, ovulation and hormones involved**

- Per Association of Professors of Gynecology & Obstetrics (APGO), what is the length of a normal cycle?

*21 to 45 days (about 1 and a half months).*

- What is the nomenclature for the first part of the ovarian phase and endometrium phase? What is the primary hormone released from the anterior pituitary and ovaries/corpus luteum during this time?

*First part of menstrual cycle corresponds with follicular phase for ovaries and proliferative phase for endometrium. The dominant hormones are follicular stimulating hormone from anterior pituitary, and estrogen from ovaries and peripheral conversion.*

- What is the nomenclature for the second part of the ovarian phase and endometrium phase? What is the primary hormone released from the anterior pituitary and corpus luteum during this time?

*The second part of the part of the menstrual cycle corresponds with the luteal phase for the ovaries, and the secretory phase for the endometrium. The primary hormone is luteinizing hormone from anterior pituitary, and progesterone from the corpus luteum.*

- What hormone surge causes ovulation to occur?

*Luteinizing hormone.*

- How long is the luteal phase?

*Fourteen days, always.*

- How long is the follicular phase?

*Varies between patients. Found by subtracting fourteen days (luteal phase) from last day of menses to the first day of the next menses.*

- When does the corpus luteum stop producing progesterone when pregnancy occurs?

*10 to 12 weeks (about 3 months) after ovulation.*

**#7: Fetal blood circulation with shunts and oxygenated/deoxygenated blood in appropriate areas, plus cross section of umbilical cord anatomy**

- Name the three active shunts in utero and then their adult remnants.

*Ductus venosus in utero to ligamentum venosum. Foramen ovale in utero to fossa ovalis. Ductus arteriosus in utero to ligamentum arteriosum.*

- What happens to the umbilical artery after birth?

*Becomes medial umbilical ligament and branch of the internal iliac artery.*

- Which important physiological changes occur to the lungs at delivery and its impact on blood circulation?

*Infant’s first inhalation causes negative pressure in lungs which leads to closure of foramen ovale.*
