# Supplementary material for: Learning Pelvic Anatomy and Pathology Through Drawing: An Interactive Session in the Obstetrics and Gynecology Clerkship
Source: MedEdPORTAL. 2023 Dec 5;19:11363. doi: 10.15766/mep_2374-8265.11363 (PMC10696139; doi:10.15766/mep_2374-8265.11363)
Supplement: Supplementary file 1 — Anatomy Presentation.pptxAnatomy Teacher Instructions.docxAnatomy Teaching Questions.docxAnatomy Teaching Questions with Answers.docxAnatomy Online Assessment.docxAnatomy Survey.docx [file mep_2374-8265.11363-s001.zip › E. Anatomy Online Assessment.docx]

**Appendix E: Anatomy online assessment**

Appendix E: Anatomy online assessment

*Utilize this appendix to create an online assessment for students who completed the anatomy interactive session. Assessment can be taken remotely and was open book. 1 hour time was given for 16 questions. We suggest that to objectively measure the effectiveness of the session to make the session closed book. You may still give the assessment remotely.*

1.
30 y/o G0 presents to the ED for the 4^th^ time in 2 years with a painful left vulvar mass described as the size of a golf ball. The patient’s records revealed a history of a Bartholin gland cyst and each time the cyst was incised and drained. A Word catheter was placed, and the patient followed up with her gynecologist where the Word catheter was removed. The ED physician requests a gynecology consult. The gynecologist finds the problem has always been on her left side. It has been present for 2 days. She finds it uncomfortable to sit, walk, and describes discomfort on a level of 2/10. She denies fever, headache, nausea, vomiting, or changes in daily bowel movements. She has been in a sexually active monogamous relationship for 5 years. Her gynecology checkup 6 months ago was normal. On exam, the mass is mobile, non-tender, and without surrounding erythema. The pelvic exam is normal, and her vital signs are within normal limits.

Which management plan is best?

a. Incision and drainage with placement of a Word Catheter

b. Prescribe a course of broad-spectrum antibiotics

c. Recommend sitz bath at home, and plan for marsupialization

d. Excise the cyst

e. Reassurance

Answer: c.

Rationale: Marsupialization is a surgical technique to cut a slit through the length of the cyst and suture the interior edge of the cyst to the exterior edge of the skin so continuous drainage can happen. Since this is a recurrent problem, eventual marsupialization is needed. A Word catheter can be used to help promote continuous drainage and encourage fistula formation, but it is temporary. Antibiotics may be used as an adjunct treatment for Bartholin's cyst abscess.

2. The most inferior border of the mons pubis is adjacent to which structure?

a. Urethra
b. Clitoris
c. Labia Minora
d. Vagina
Answer: b.

Rationale: The clitoris is the most anterior structure of the labia. The first opening under the clitoris is the urethra, then vagina. Sometimes the urethra is just inside the vagina. The apices of the labia minora form the clitoral hood anteriorly which is the most adjacent structure to the mons pubis.

3. Which statement best describes the uterine artery?

a. The uterine artery is a branch of the external iliac artery.
b. As the uterine artery enters the uterus, it passes posterior to the ureters.

c. The uterine artery anastomoses with the ovarian artery bilaterally.
d. The uterine artery is contained within the uterosacral ligament.
Answer: c.

Rationale: The uterine artery is a branch of the internal iliac artery, and the external iliac artery goes into the groin and down the leg. At the pelvic brim, the ureter passes above common iliac, but as it dives into the pelvis it passes under the uterine artery (water under the bridge). The uterine artery does enter at the cervix-uterine junction, but the uterine artery passes anterior to the ureter (water under the bridge). The cardinal ligament contains the uterine artery. The ovarian and uterine artery anastomose bilaterally on the side of the uterus and create a mass of small vessels that are difficult to clamp and tie off at a hysterectomy.

4. The endometrium is made of three (3) layers. Which layer does not undergo conformational change during the menstrual cycle?

a. Stratum basalis
b. Stratum compactum
c. Stratum spongiosum
Answer: a.

Rationale: The stratum functionalis (stratum compactum + stratum spongiosa) responds to hormonal changes and appropriately sheds and grows during the menstrual cycle. The stratum basalis does not change but regenerates the other layers of the endometrium.

5. The 'classical incision' performed to accomplish a cesarean delivery is associated with which increased risk if a subsequent pregnancy were to occur?

a. Uterine rupture
b. Fetal intrauterine growth restriction
c. Placenta previa
d. Cornual ectopic
Answer: a.

Rationale: A low transverse incision is made right above the cervix, while a classic incision is made at the uterine body. In a subsequent pregnancy a low transverse uterine incision can reopen or rupture in 1/100 pregnancies, occurring most often in the lower uterine segment composed of fascia. In a classical incision up to 4-9/100 can rupture because it is made through the muscles – the contractile portion of the uterus.

6. In the case of post-partum hemorrhage, several medications such as uterotonics, vasoconstrictors, and antifibrinolytics are used to control the bleeding. If these medications do not work, a large balloon tamponade is placed within the uterus. If this does not work, an option to control hemorrhage while preserving the uterus would be surgical ligation or radiological embolization of which parent arteries bilaterally?

a. Common iliac
b. Vesicle arteries
c. Internal iliac
Answer: c.

Rationale: The internal iliac arteries are the parent arteries of the uterine arteries and are the major blood supply of the pelvic structures.

7. The ovarian vein drainage system is not the same bilaterally. Which vessel does the left ovarian vein drain into?

a. Left renal vein
b. Vena cava
c. L~~e~~ft internal iliac vein
d. L~~e~~ft common iliac vein
Answer: a.

Rationale: The l~~e~~ft ovarian vein drains into the left renal vein. The right ovarian vein drains into IVC. Both ovarian arteries stem from the aorta.

8. Laparoscopic and robotic surgery requires placement of sharp trocars through the abdominal wall. Which vessel in the abdominal wall would be at risk of injury during trocar placement?

a. Inferior epigastric artery
b. Common iliac artery
c. Internal iliac artery
d. External iliac artery
Answer: a.

Rationale: The inferior epigastric arteries are often more superficial in the abdominal wall where the others are deep in the pelvis. The abdominal aorta sits under the umbilicus just on top of the sacrum and is a retroperitoneal vessel. The patient should be placed flat and not in Trendelenburg, or it could easily be injured.

9. The spiral arteries are found in which structure?

a. Ovarian cortex
b. Vaginal muscularis
c. Endocervix
d. Endometrium
Answer: d.

Rationale: Spiral arteries are found in the endometrium and constrict and dilate as the menstrual cycle progresses. They are also a major blood supply for the placenta during pregnancy. Dysfunction of the spiral arteries during pregnancy is thought to contribute to the pathophysiology behind pre-eclampsia.

10. If a woman has a vaginal hysterectomy (via the vaginal route), in which order are the ligaments cut?

a. Uterosacral, cardinal, round
b. Round, uterosacral, cardinal
c. Round, cardinal, uterosacral
d. Cardinal, uterosacral, round
Answer: a.

Rationale: Vaginally, the uterosacral ligaments are encountered first, followed by the cardinal ligaments attaching to the cervix, and lastly the round ligaments connected to the uterine fundus. During an abdominal hysterectomy, the ligaments are encountered in the reverse order of a vaginal hysterectomy. Steps for a vaginal hysterectomy are as follows: local anesthesia is infiltrated around the cervix (front and back, not laterally). A circumferential incision is made around the cervix. The bladder is dissected off the cervix and reflected upwards. The anterior peritoneum is opened by cutting the utero-vesical peritoneal fold. The Pouch of Douglas is opened. The uterosacral ligaments are ligated and tied. The uterine arteries in the cardinal ligament are ligated and tied. The round ligaments are ligated and tied. The tubes and ovaries may be taken in this pedicle or may be preserved. The uterus and cervix are removed. The vagina is normally closed, or the edges are sutured.

11. Prolapse of the vagina can occur after hysterectomy. Areas prone to prolapse are the anterior wall (cystocele), posterior wall (rectocele) and apex (apical vault prolapse). To prevent apical vault prolapse at the time of hysterectomy the gynecologist ensures that 2 ligaments previously attached to the uterus are sutured to or incorporated into the closure of the vaginal cuff. Which ligaments are being referred to here?

a. Infundibulopelvic ligaments
b. Cardinal ligaments
c. Uterosacral ligaments
d. Round ligaments
Answer: c.

Rationale: The surgeon will place stitches through the open vaginal cuff and attach them to the uterosacral ligaments bilaterally. Those ligaments do not have a large blood supply and come from the sacrum, high in the pelvis so that the vagina stays higher and does not have the propensity to prolapse.

12. Sampson's artery is a vessel contained within which ligament?

a. Infundibulopelvic
b. Cardinal
c. Uterosacral
d. Round
Answer: d.

Rationale: The round ligament carries Sampson’s artery. Cardinal ligament carries uterine vessels. The infundibulopelvic ligament carries ovarian vessels. The uterosacral ligaments do not carry any arteries or vessels.

13. Which ligaments contain the ovarian blood supply?

a. Infundibulopelvic
b. Cardinal
c. Uterosacral
d. Round
Answer: a.

Rationale: The infundibulopelvic ligament carries ovarian vessels that originate from the ovarian vessel off the abdominal aorta. If the vessel is not correctly grasped and ties, it recedes back to the abdominal aorta and a large amount of blood can be lost quickly.

14. Which of the following statements are not true about the labia minora and the labia majora?

a. The labia majora contain hair follicles while the labia minora do not
b. The labia majora contain more adipose tissue than the labia minora
c. The apices of the labia majora form the clitoral hood
d. The labia minora vary in size, symmetry, and shape more than the labia majora
Answer: c.

Rationale: The apices of the labia minora form the clitoral hood.

15. Two centimeters superior to the mons pubis is an area used for an 8-10 cm horizontal incision to accomplish many gynecologic, obstetric, and urologic surgeries. What is the name of this incision?

a. Low transverse
b. Maylard
c. McBurney's
d. Pfannenstiel
Answer: d.

Rationale: The low transverse incision is much higher than the Pfannenstiel that is used for most all gynecology and obstetrics procedures. The Maylard incision is 2 cm below the umbilicus transversely. The McBurney incision over McBurney point is at a diagonal in the right lower quadrant.

16. The perineal body is the triangular central tendon of the perineum. This fibromuscular mass is created by the convergence of several muscles. Which muscle does not have direct attachments to the perineal body?

a. External anal sphincter
b. Bulbocavernosus
c. Transverse perineal
d. Ischiocavernosus
Answer: d.

Rationale: The ischiocavernosus muscles go from the pubic bone to the ischium, not close to the anus. The transverse perineal muscle runs from the ischial tuberosity through the perineal body. The anal muscle is around the rectum and attaches into the perineal body. It often can tear during vaginal delivery, especially with an episiotomy. The bulbocavernosus muscle goes from the pubic bone to the perineal body.
