# Supplementary material for: Learning Pelvic Anatomy and Pathology Through Drawing: An Interactive Session in the Obstetrics and Gynecology Clerkship
Source: MedEdPORTAL. 2023 Dec 5;19:11363. doi: 10.15766/mep_2374-8265.11363 (PMC10696139; doi:10.15766/mep_2374-8265.11363)
Supplement: Supplementary file 1 — Anatomy Presentation.pptxAnatomy Teacher Instructions.docxAnatomy Teaching Questions.docxAnatomy Teaching Questions with Answers.docxAnatomy Online Assessment.docxAnatomy Survey.docx [file mep_2374-8265.11363-s001.zip › F. Anatomy Survey.docx]

**Appendix F: Anatomy survey**

Appendix F: Anatomy survey

*Faculty Instructions: Anatomy survey should be given during the Ob/Gyn clerkship to third-year medical students. Question #5 is intended to be free response. Utilize the information from this survey to gage anatomy interactive session learning content for subsequent clerkship groups. It should take 5 minutes to complete.*

Student instructions: Complete this survey during the last week of your Ob/Gyn third-year clerkship. Circle ‘Yes’ or ‘No’ for question #1-4. For question #5, write within the textbox your answer to the question.

| **Anatomy Interactive Session Survey** |  |  |
| --- | --- | --- |
| 1. Prior to the OB/GYN anatomy session, I had a thorough understanding of pelvic anatomy. | Yes | No |
| 1. After the OB/GYN anatomy session, I had a thorough understanding of pelvic anatomy. | Yes | No |
| 1. Did the anatomy session prepare you for the surgical portions of the rotation? | Yes | No |
| 1. Has drawing the pelvic structures helped your understanding of the pelvic pathology? | Yes | No |
| 1. How do you know drawing pelvic structures has helped your understanding of pelvic pathology? (Free response) |  |  |
